# Performance and labor conditions of physiotherapists in Brazilian intensive care units during the COVID-19 pandemic. What did we learn?

**DOI:** 10.5935/2965-2774.20230359-en

**Published:** 2023

**Authors:** Andressa Sabrina de Oliveira Resende, Suzi Laine Longo dos Santos Bacci, Ítalo Ribeiro Paula, Leandro Alves Pereira, Cíntia Johnston, Valéria Cabral Neves Luszczynski, Vívian Mara Gonçalves de Oliveira Azevedo

**Affiliations:** 1 Multiprofessional Residency in Child Health Care, Faculdade de Medicina, Universidade Federal de Uberlândia - Uberlândia (MG), Brazil; 2 Hospital de Clínicas de Uberlândia, Empresa Brasileira de Serviços Hospitalares, Universidade Federal de Uberlândia - Uberlândia (MG), Brazil; 3 Faculdade de Matemática, Universidade Federal de Uberlândia - Uberlândia (MG), Brazil; 4 Department of Pediatrics, Faculdade de Medicina, Universidade de São Paulo - São Paulo (SP), Brazil; 5 Hospital de Clínicas, Universidade Federal do Paraná - Curitiba (PR), Brazil; 6 Faculdade de Educação Física e Fisioterapia, Universidade Federal de Uberlândia - Uberlândia (MG), Brazil

**Keywords:** COVID-19, Physiotherapists, Survey and questionnaires, Occupational risks, Professional training, Work hours, Remuneration, Intensive care units

## Abstract

**Objective:**

To describe the role of physiotherapists in assisting patients suspected to have or diagnosed with COVID-19 hospitalized in intensive care units in Brazil regarding technical training, working time, care practice, labor conditions and remuneration.

**Methods:**

An analytical cross-sectional survey was carried out through an electronic questionnaire distributed to physiotherapists who worked in the care of patients with COVID-19 in Brazilian intensive care units.

**Results:**

A total of 657 questionnaires were completed by physiotherapists from the five regions of the country, with 85.3% working in adult, 5.4% in neonatal, 5.3% in pediatric and 3.8% in mixed intensive care units (pediatric and neonatal). In intensive care units with a physiotherapists available 24 hours/day, physiotherapists worked more frequently (90.6%) in the assembly, titration, and monitoring of noninvasive ventilation (p = 0.001). Most intensive care units with 12-hour/day physiotherapists (25.8%) did not apply any protocol compared to intensive care units with 18-hour/day physiotherapy (9.9%) *versus* 24 hours/day (10.2%) (p = 0.032). Most of the respondents (51.0%) received remuneration 2 or 3 times the minimum wage, and only 25.1% received an additional payment for working with patients suspected to have or diagnosed with COVID-19; 85.7% of them did not experience a lack of personal protective equipment.

**Conclusion:**

Intensive care units with 24-hour/day physiotherapists had higher percentages of protocols and noninvasive ventilation for patients with COVID-19. The use of specific resources varied between the types of intensive care units and hospitals and in relation to the physiotherapists’ labor conditions. This study showed that most professionals had little experience in intensive care and low wages.

## INTRODUCTION

In December 2019 in China, contagious respiratory infections caused by an unknown virus were reported. After several studies,^(1-3)^ the etiology of this disease was found to be attributed to a new virus belonging to the *Coronaviridae* family, severe acute respiratory syndrome coronavirus 2 (SARS-CoV-2). This virus, capable of infecting animals and humans with rapid transmission, caused the coronavirus disease 2019 (COVID-19) pandemic, declared by the World Health Organization (WHO), in February 2020.^(2,3)^

In Brazil, according to the Ministry of Health, from February 2020 to early June 2022, the number of confirmed cases was 31.153.069, with 666.997 deaths. There was a reduction in the number of cases due to vaccination; however, currently, there is an increase in the number of cases. In 2020, Brazil became the epicenter of this disease.^(4)^

COVID-19 causes fever and respiratory symptoms such as dry cough, fatigue, dyspnea, and severe pneumonia. The diagnosis is clinical and confirmed by laboratory tests.^(3)^ For its treatment, focusing on the integral care of the patient and the quality of care, a multiprofessional team and various intervention and treatment strategies are necessary.^(5)^

Physiotherapists are frontline professionals against COVID-19, providing assistance with the aim of improving overall functionality and the quality of life of patients. Their main role is the prevention, assistance and recovery of these seriously ill individuals and the continuity of care after hospital discharge. However, studies that investigated the role and labor conditions of physiotherapists at the beginning of the COVID-19 pandemic in Brazil are scarce.^(6)^

Knowing that physiotherapists have an important role in intensive care units (ICUs) and that at the beginning of the pandemic, there was a disproportionate relationship between the growing demand of patients and the availability of ideal resources for treatment, it is necessary to evaluate the labor conditions of these professionals and the opportunities for technical qualification, as the pandemic was an unprecedented situation.

Therefore, the main objective of this study was to describe the role of physiotherapists in assisting patients with suspected or diagnosed COVID-19 hospitalized in ICUs in Brazil regarding technical training, working time, care practice and labor conditions. In addition, we compared the time during which the physiotherapist worked in the ICU with the use of noninvasive ventilation (NIV) and use of protocols; the different types of ICUs in relation to the risk of aerosolization, use of NIV, application of protocols and staff training; and the types of hospitals and protection strategies, training, types of humidification, and participation of the physiotherapists in committees and decisions.

## METHODS

This was an analytical cross-sectional survey carried out through an electronic questionnaire distributed to physiotherapists who worked in the care of individuals with suspected or confirmed COVID-19 in ICUs in Brazil. This study was approved by the Ethics Committee for Research with Human Beings of the institution involved (no. 4.447.861) and by the Clinical Research Committee of the *Associação de Medicina Intensiva Brasileira* (AMIB, AMIB-net).

For the sample size calculation, we considered 10% of the number of physiotherapists registered at AMIB (total of 5,000) who worked in neonatal, pediatric, and mixed (neonatal and pediatric) ICUs and adult ICUs. Thus, the estimated minimum sample size was 500 survey participants.

As this was a nationwide study that sought to reach the majority of physiotherapists who worked directly in Brazilian ICUs, a link to access the electronic questionnaire was sent through email, phone messages and social networks (nonrandom sampling strategy, i.e., convenience sampling). Free and Informed Consent Forms, to clarify the possible risks and benefits of the research, were made available to the participants, along with the link to access the questionnaire.

The electronic questionnaire was prepared by physiotherapists with expertise in intensive care and who worked in referral hospitals for the care of patients with suspected or confirmed COVID-19. Google Forms®, which has online and free access, was used to prepare the questionnaire. A pilot test was carried out with 14 respondents to evaluate the adequacy of the questionnaire before being sent to the physiotherapists in the sample of this study.

The instrument contained a total of 55 questions divided into 6 sessions, covering the following themes: participant characteristics, ICU characteristics, data regarding training and physiotherapy care protocols, use of personal protective equipment (PPE) available to professionals, and aspects of the physiotherapist’s clinical care performance in this context (Supplementary Material).

The collected data were tabulated in a Microsoft Office Excel version 2007 spreadsheet, with quantitative variables presented as percentages and frequencies, taking into account the nature and specificity of the data. As the variables shown were categorical, Fisher’s exact test was used to compare the time during which the physiotherapists worked in the ICU with the use of NIV and use of protocols; the types of ICUs and risk of aerosolization, use of NIV, application of protocols and staff training; and the types of hospitals and protection strategies, training, types of humidification, committees, and decisions. The analyses were performed using R software (R Core Team, 2015), and for all analyses, p values < 0.05 were considered statistically significant.

## RESULTS

A total of 657 responses from physiotherapists from all Brazilian states were analyzed. In 79% of the hospital’s physiotherapists worked exclusively in the ICU, and in 66.2% of the ICUs, the total number of beds was from 10 to 20; in 80.0% of the hospitals, there was a physiotherapists responsible for 6 to 10 beds. Table 1 shows the distribution of respondents according to the region of the country, the role of the participating physiotherapists, working time, type of ICU and type of hospital in which they worked.

**Table 1:** Sample characteristics

Variables	n (%)
Regions	
North	31 (4.7)
Northeast	124 (18.8)
South	180 (27.4)
Southeast	269 (40.9)
Midwest	53 (8.0)
Position of the physiotherapists^*^	
On duty	365 (55.5)
General coordinator	43 (6.5)
Daily worker	159 (24.2)
Unit coordinator	22 (3.3)
Work experience (years)	
Less than 1	140 (21.3)
Between 1 and 5	222 (33.7)
Between 6 and 10	114 (17.3)
More than 10	181 (27.5)
Types of ICUs	
Adult	561 (85.3)
Neonatal	36 (5.4)
Pediatric	35 (5.3)
Mixed	25 (3.8)
Types of hospitals	
Public	208 (31.6)
Private	206 (31.3)
University	179 (27.2)
Philanthropic	58 (8.8)
Military	6 (0.9)

ICU - intensive care unit.

* 68 participants did not answer this question.

The comparison between the time the physiotherapists worked in the ICU and the use of NIV and protocols is presented in table 2. Noninvasive ventilation and protocols were used more frequently in units that had 24-hour/day physiotherapy (p = 0.006; p = 0.032, respectively).

**Table 2:** Comparison between the time the physiotherapists worked in the intensive care unit and the application of noninvasive ventilation and use of protocols

Variables	Physiotherapist performance time (per day)				p value
	12 hours/day (n = 58)	18 hours/day (n = 121)	24 hours/day (n = 461)	Others (n = 17)	
Received NIV (%)					0.006
After extubation	1.7	3.3	1.9	0	
After viral reduction	3.5	3.3	3.9	5.9	
At any time	77.6^A^	90.1^AB^	90.9^B^	82.3^AB^	
No	15.5^A^	1.7^B^	2.4^B^	11.8^AB^	
Unsure	1.7	1.6	0.9	0	
NIV (%)					0.001
Equipment assembly	5.2	9.9	5.4	0	
Equipment assembly, titration and monitoring of parameters	77.6^A^	84.3^AB^	90.7^B^	82.3^AB^	
Titration and monitoring of parameters	8.6	4.2	3.3	5.9	
Did not act	1.7	0.8	0.4	5.9	
Did not answer	6.9	0.8	0.2	5.9	
Protocols (%)					0.032
Physiotherapists specific	6.9	9.9	12.6	29.4	
Multiprofessional team	34.5	34.7	29.3	23.5	
Multiprofessional team and physiotherapists	29.3	41.3	44.2	23.5	
None	25.9^A^	9.9^B^	10.2^B^	17.7^AB^	
Unsure	3.4	4.2	3.7	5.9	

NIV - noninvasive ventilation. Proportions in the rows followed by the same superscript letter did not differ significantly from each other by the multiple comparison test, considering a significance level of 5% (p < 0.05). The rows that do not have superscript letters did not differ significantly.

We compared the different types of ICUs in relation to the risk of aerosolization, use of NIV, application of protocols and staff training. The results showed that even at the beginning of the pandemic, NIV was used for patients of all ages, with different types of interfaces. Pediatric ICUs used more heat and moisture exchangers (HMEs) plus high-efficiency particulate air (HEPA) filters than other types of ICUs (p < 0.001). The other results are presented in table 3.

**Table 3:** Comparison between intensive care units serving different age groups regarding the risk of aerosolization, use of noninvasive ventilation, application of protocols and staff training in Brazil

Variables	Adult ICU n = 561 n = 25	Mixed ICU n = 36	Neonatal ICU n = 35	Pediatric ICU	p value
Use of NIV (%)					< 0.001
After extubation	1.6	4	2.8	8.6	
After viral reduction	3.9	0	2.8	5.7	
At any time during hospitalization	91.8^A^	88^AB^	72.2^B^	68.6^B^	
Did not apply NIV	2.2^A^	4^AB^	19.4^B^	11.4^B^	
Did not answer	0.5	4	2.8	5.7	
Interface type (%)					< 0.001
Ventilated/nonventilated face mask	24.4^A^	12^AB^	0^B^	8.6^AB^	
Full face, nasal and/or full face mask	29.1^A^	32^A^	5.6^B^	22.9^AB^	
Full face mask (ventilated or not)	16.9	12	2.8	11.4	
Masks (facial, nasal, full face) and helmet	22.3^A^	0^B^	0^B^	5.7^B^	
Masks (facial, nasal, full face) and nasal prongs	2.8^A^	36^B^	19.4^B^	34.3^B^	
Masks, helmet and nasal prong	3.1	0	0	5.7	
Nasal prongs	1.4^A^	8^AB^	72.2^C^	11.4^B^	
Humidification type (%)					< 0.001
HME	18.2	0	5.6	2.9	
HME + HEPA	48.3^A^	28^B^	16.7^B^	71.4^C^	
HMEF	32.3	32	11.1	14.3	
AH + HEPA	1.2^A^	40^C^	66.6^C^	11.4^B^	
Participation in intubation (%)					< 0.001
Yes	97.5^A^	96^AB^	91.7^AB^	88.6^B^	
No	0^A^	0^AB^	5.5^B^	8.6^B^	
Sometimes	2.5	4	2.8	2.8	
Airway clearing interventions (%)					0.023
Yes	17.6	4	16.7	2.9	
No	82.4	96	83.3	97.1	
Early mobilization (%)					0.017
Yes	88.6^A^	88^AB^	72.3^B^	97.1^A^	
No	11.4^A^	12^AB^	27.7^B^	2.9^A^	
Protocol (%)					0.001
Physiotherapists specific	13.0	20	0	2.9	
Multiprofessional team	27.8^A^	36^A^	66.6^B^	34.3^A^	
Multiprofessional team and physiotherapist	42.6	32	27.8	51.4	
None	12.7	8	5.6	5.7	
Unsure	3.9	4	0	5.7	
Training (%)					0.110
Yes	69.4	80	86.2	74.3	
No	30.6	20	13.8	25.7	
Type of training (%)					< 0.001
Alone	7.5	0	5.5	5.7	
Online	5.7^A^	40^B^	5.5^A^	17.1^A^	
Presential	34.6	16	33.4	20	
In person and online	20.7^A^	24^AB^	41.7^B^	22.9^AB^	
Others	0.9	0	0	8.6	
Had no training	30.6^A^	20^AB^	13.9^B^	25.7^AB^	
Closed suction circuit (%)					0.176
Yes	96.7	96	91.7	94.3	
No	3.3	4	8.3	5.7	

ICU - intensive care unit; NIV - noninvasive ventilation; HME - heat and moisture exchanger; HEPA - high efficiency particulate air; HMEF - heat and moisture exchanger filter; AH - active humidification. The proportions followed by the same superscript letters (in the lines) did not differ significantly from each other in the test of multiple comparisons of proportions with Bonferroni adjustment, with p < 0.05 being considered significant by Fisher's exact test. Lines that do not have superscript letters did not show significant differences by Fisher's exact test.

Regarding the different types of hospitals, the availability of PPE for the team was not significantly different (p = 0.569). University and private hospitals used beds with negative pressure more frequently (13.4% and 17%, respectively) than public hospitals (9.3%) (p = 0.018). The results also showed that university hospitals trained their teams more frequently (79.3%) than public hospitals (65.8%, p = 0.020), but there was no difference in relation to the other types of hospitals (Table 4).

**Table 4:** Comparison between type of hospitals and protection strategies, training, types of humidification, and participation in decision committees in Brazil

Variables	Types of hospitals					p value
	Philanthropic n = 58	Military n = 6	University n = 179	Private n = 206	Public n = 208	
Negative pressure room (%)						0.018
Yes	22.4^AB^	16.7^AB^	30.7^A^	27.2^A^	16.8^B^	
No	77.6^AB^	83.3^AB^	69.3^A^	72.8^A^	83.2^B^	
PPE for the whole team (%)						0.056
Yes	87.9	83.4	86.0	88.4	82.3	
No	0	0	1.1	0.5	1.4	
Most of the time	10.4	16.6	11.8	10.2	11.0	
Rarely	0	0	0	0	0.9	
Not all necessary	1.7	0	1.1	0.9	4.4	
Received training (%)						0.020
Yes	72.6^AB^	50^AB^	79.3^A^	68.9^AB^	65.9^B^	
No	27.4^AB^	50^AB^	20.7^A^	31.1^AB^	34.1^B^	
Humidification type (%)						< 0.001
HME	18.9^AB^	33.3^AB^	12.3^A^	11.1^A^	22.6^B^	
HME + HEPA	37.9^AC^	66.7^ABC^	49.7^AB^	57.5^B^	36.5^C^	
HMEF	39.7^A^	0^AB^	30.7^AB^	22.2^B^	35.6^A^	
AH + HEPA	3.5	0	7.3	9.2	5.3	
Closed suction circuit (%)						0.003
Yes	98.3^A^	66.7^B^	98.3^A^	97.6^A^	93.8^AB^	
No	1.7^A^	33.3^B^	1.7^A^	2.4^A^	6.2^AB^	
Participation in decision committees (%)						< 0.001
Yes	58.6^AB^	16.7^A^	68.7^B^	47.1^A^	44.2^A^	
No	41.4^AB^	83.3^A^	31.3^B^	52.9^A^	55.8^A^	

PPE - personal protective equipment; HME - heat and moisture exchanger; HEPA - high efficiency particulate air; HMEF - heat and moisture exchanger filter; AH - active humidification. Proportions in the rows followed by the same superscript letter did not differ significantly from each other by the multiple comparison test, considering a significance level of 5% (p < 0.05). The rows that do not have superscript letters did not differ significantly.

Some variables, such as the use of high-flow nasal cannula (HFNC) oxygen therapy, the participation of the physiotherapists in the intubation process and the risk of aerosolization, did not present a statistically significant difference between the hospitals.

Regarding remuneration, most of the responding physiotherapists (51.0%) received two or three times the minimum wage. Regarding additional payment, 66.7% of physiotherapists received additional hazard pay, and only 25.11% received a maximum payment for working with individuals with suspected or confirmed COVID-19. Although 66.8% were specialized, only 48.7% had a specialist title recognized by the *Conselho Federal de Fisioterapia e Terapia Ocupacional* (COFITTO).

## DISCUSSION

Due to the need to increase the number of physiotherapists in ICUs during the COVID-19 pandemic, this survey showed a greater number of professionals with one to five years of experience and low salaries. In addition, most participants did not receive a maximum payment for working during the pandemic.

Hazard pay was granted for specific work activities and could be suppressed or reduced in the face of evidence of elimination or reduction of exposure to the worker through the use of PPE. This situation, called conditional salary, supports the employer in granting or not granting the maximum hazard pay, as seen in article 191, II of the labor laws and in the regulatory norm (NR-15).^(7,8)^

The present study showed that early mobilization was more frequent in pediatric ICUs, but with no significant difference in relation to adult and mixed units. Mobilization and physical exercise protocols must observe each stage of the disease and the patient’s clinical condition to promote beneficial effects and not cause adverse effects.^(9)^

Intensive care physiotherapists were among the health professionals involved in the care of patients with COVID-19 and played a key role in the management of postural changes, early mobilization, management of noninvasive support, and weaning from invasive mechanical ventilatory support.^(10)^ In this way, we emphasized the importance of the physiotherapist’s performance in the management and care of patients with COVID-19.

This survey showed that the protocols were more frequent in ICUs with a physiotherapist available 24 hours/day. Studies^(11-13)^ report that the implementation and application of protocols help standardize care, increasing the involvement of the multiprofessional team and improving the quality and safety of care. Previous studies^(14-16)^ related a higher frequency of use of protocols in the presence of physiotherapists in ICUs.

Our results also showed that ICUs with a 24-hour/day physiotherapists used NIV more frequently, with this professional being responsible for its assembly, titration and monitoring in all types of ICUs. Previous surveys reinforced that mechanical ventilation protocols were more available in ICUs whose physiotherapists had exclusive or shared responsibility for managing ventilatory support.^(11,17)^

The application of NIV and HFNC, as well as endotracheal aspiration and intubation procedures, increase the risk of aerosol production and environmental contamination.^(18)^ The use of a negative pressure room and the proper use of PPE are recommended during procedures with aerosol generation.^(19)^ However, the results of this survey showed that most hospitals did not use a negative pressure room.

The risk of aerosolization, especially in rooms with suspected or confirmed COVID-19 patients, can be mitigated through the use of passive humidification filters.^(20)^ However, the type of humidification chosen depends on the effects it will have on the patient, such as the estimate of dead space, increased airway resistance and efficiency in the humidification provided to the patient.^(21-23)^ For pediatric patients, it is necessary to consider the patient’s weight and tidal volume when choosing the type of filter.^(17)^ In this survey, active humidification associated with HEPA filters was the most commonly used in neonatal and mixed ICUs. On the other hand, the use of HME + HEPA filters was higher in pediatric ICUs compared to other types of ICUs in university hospitals and private hospitals compared to public hospitals.

The use of NIV and HFNC at the beginning of the pandemic was strongly restricted due to the risk of aerosolization with these resources, and earlier endotracheal intubation was recommended.^(24)^ However, recent evidence has demonstrated the need to use these therapies for the treatment of respiratory failure, as long as they are used correctly, following the indications and taking care to avoid the spread of aerosols using the most viable interface and the appropriate PPE.^(25)^ The use of NIV significantly reduces hospital mortality rates, intubation and length of stay among patients with COVID-19.^(26)^ Nevertheless, the incorrect use of these resources can also cause damage and even death in severe cases.^(27,28)^

Although we have passed the peak of the pandemic, this study is important because it describes the labor conditions of physiotherapists and the current performance, working time and remuneration of these professionals. The presence of a physiotherapist 24 hours/day has shown important results in clinical practice.

The main limitation of this study is that it was a digital survey using a single database and a nonrandom sampling strategy (convenience sampling). As the exact number of physiotherapists in Brazil working in ICUs is not known, the number of professionals may be greater than expected. Moreover, the physiotherapists included in this study may be those who work in reference centers, in states with greater incomes, and closest to AMIB society courses and congresses. Physiotherapists with AMIB membership were more easily reached through e-mail, phone, and social media than physiotherapists working in regions with smaller incomes and distances from larger reference hospitals. This might explain the low percentage of physiotherapists who experienced a lack of PPE in this survey and might have overestimated the wages and prevalence of specialization among professionals.

## CONCLUSION

Intensive care units with physiotherapists 24 hours/day had higher percentages of protocols and noninvasive ventilation among patients with COVID-19 and greater professional autonomy regarding the use of specific physiotherapy protocols and noninvasive ventilation management. The use of specific resources varied between the types of intensive care units and hospitals and in relation to the physiotherapists’ labor conditions. This study showed that most professionals had little experience in intensive care and low wages.

More studies are needed to describe the role of physiotherapists and their labor conditions in the face of health crises.
